# Expression Patterns of Extracellular Matrix Proteins during Posterior Commissure Development

**DOI:** 10.3389/fnana.2016.00089

**Published:** 2016-09-28

**Authors:** Karen Stanic, Natalia Saldivia, Benjamín Förstera, Marcela Torrejón, Hernán Montecinos, Teresa Caprile

**Affiliations:** ^1^Axon Guidance Laboratory, Department of Cell Biology, Faculty of Biological Sciences, University of ConcepciónConcepción, Chile; ^2^Department of Physiology, Faculty of Biological Sciences, University of ConcepciónConcepción, Chile; ^3^Department of Biochemistry and Molecular Biology, Faculty of Biological Sciences, University of ConcepciónConcepción, Chile

**Keywords:** posterior commissure, SCO-spondin, axon guidance, extracellular matrix, prosomere 1, laminin, osteopontin, tenascin

## Abstract

Extracellular matrix (ECM) molecules are pivotal for central nervous system (CNS) development, facilitating cell migration, axonal growth, myelination, dendritic spine formation, and synaptic plasticity, among other processes. During axon guidance, the ECM not only acts as a permissive or non-permissive substrate for navigating axons, but also modulates the effects of classical guidance cues, such as netrin or Eph/ephrin family members. Despite being highly important, little is known about the expression of ECM molecules during CNS development. Therefore, this study assessed the molecular expression patterns of tenascin, HNK-1, laminin, fibronectin, perlecan, decorin, and osteopontin along chick embryo prosomere 1 during posterior commissure development. The posterior commissure is the first transversal axonal tract of the embryonic vertebrate brain. Located in the dorso-caudal portion of prosomere 1, posterior commissure axons primarily arise from the neurons of basal pretectal nuclei that run dorsally to the roof plate midline, where some turn toward the ipsilateral side. Expressional analysis of ECM molecules in this area these revealed to be highly arranged, and molecule interactions with axon fascicles suggested involvement in processes other than structural support. In particular, tenascin and the HNK-1 epitope extended in ventro-dorsal columns and enclosed axons during navigation to the roof plate. Laminin and osteopontin were expressed in the midline, very close to axons that at this point must decide between extending to the contralateral side or turning to the ipsilateral side. Finally, fibronectin, decorin, and perlecan appeared unrelated to axonal pathfinding in this region and were instead restricted to the external limiting membrane. In summary, the present report provides evidence for an intricate expression of different extracellular molecules that may cooperate in guiding posterior commissure axons.

## Introduction

While the extracellular matrix (ECM) has historically been viewed as an inert supportive mesh for tissue, it is now known that the ECM plays various roles in signaling and modulation from the first developmental stages until adulthood (Zimmermann and Dours-Zimmermann, 2008). These functions can be carried out either directly through the binding of ECM components to cellular receptors, thus triggering or influencing signaling events across the cell membrane, or indirectly through anchoring signaling factors to regulate respective bioavailability. The ECM also fulfills mechanical roles, such as providing structural support by forming barriers and filters, as well as generating microdomains with different cell adhesion capacities (Dityatev et al., 2010).

Functional *in vitro* studies and genetic analyses evidence that the ECM affects virtually all aspects of central nervous system (CNS) development, impacting, for example, cell migration, axonal growth, myelination, dendritic spine formation, and synaptic plasticity (Barros et al., 2011). Long-standing research exists regarding the role of the ECM during axonal growth and guidance (e.g., as reviewed in Westerfield, 1987). In particular, *in vitro* studies demonstrate the effects that different ECM components have as substrates for embryonic neurons, knowledge supported in recent years through analysis with mutant animals (Barros et al., 2011). Indeed, the highly reproducible patterns of axonal outgrowth in developing embryos imply that axons are actively guided by conserved information in the surrounding environment. This information is sensed by the axonal growth cone, highly motive tip of the growing axons, that responds by the reorganization and dynamics of the actin and microtubule cytoskeleton with the consequent advance, retraction, or axonal turn (Kalil and Dent, 2005).

Axon guidance cues exist in diffusible (i.e., netrins, f-spondin, and slit) or cell surface-associated (i.e., semaphorins, Eph, and ephrins) forms that regulate long- or short-range axon guidance, respectively (Chilton, 2006; Nawabi and Castellani, 2011). However, it is important to consider that these guidance cues are immersed in a highly intricate and changing ECM, which can influence axonal effects. Related to this, several *in vitro* studies report that ECM components can generate a permissive or non-permissive substrate for navigating axons and can modulate the effects of classical guidance cues, such as of netrin or Eph/ephrin family members (Hopker et al., 1999; Suh et al., 2004). The ECM molecules bind and signal through integrin transmembrane receptors, heterodimeric proteins composed by α and β subunits. The interaction of integrins to their ligands regulates cytoskeletal dynamics, through associated proteins such as talin, vinculin, integrin-linked kinase (ILK), or focal adhesion kinase (FAK), controlling the growth cone adhesion and assembly of the actin and microtubule cytoskeletons (Nakamoto et al., 2004; Myers et al., 2011). Despite the noted importance of ECM molecules, little knowledge exists regarding the expression of these components during CNS development.

Bilaterally symmetrical organisms need to exchange information between each side of the body to integrate sensory inputs and coordinate motor control. This exchange occurs through commissures formed by neurons that project axons across the midline to the contralateral side of the CNS (Dickson and Zou, 2010; Nawabi and Castellani, 2011). On the encephalic level, the first transversal commissure to develop is the posterior commissure (PC), a conspicuous decussation of fibers originating mainly in pretectal nuclei and serving auxiliary visual functions (Mastick and Easter, 1996). The PC crosses the midline through the dorsal portion of prosomere 1, and the caudal border of this commissure corresponds to the diencephalic-mesencephalic boundary (Puelles and Rubenstein, 2003; Ferran et al., 2007). The PC is highly conserved along the vertebrates. In anamniotes (e.g., cat shark and zebrafish) the PC axons originate from two populations of neurons located at the diencephalic-mesencephalic boundary, one of them located dorsally and the other ventrally (Ware et al., 2015). In amniotes (e.g., chick and mouse), PC axons principally originate from magnocellular nucleus neurons of the PC located at the ventrolateral pretectum, which run dorsally to the midline. To a lesser extent, the PC axons also arise from parvocellular nucleus neurons of the PC that project to the dorsal midline to reach and cross the roof plate (Mastick and Easter, 1996; Ferran et al., 2007, 2009; Merchan et al., 2011; Ware and Schubert, 2011; Ware et al., 2015).

The trajectory of PC axons can be divided into three stages. First, the axons run dorsally toward the lateral roof plate. In this step, studies performed in chick embryos revealed that axons are surrounded by EphA7-expressing cells that appear to form a repulsion barrier that delimits the axonal trajectory (Stanic et al., 2014). Second, in the lateral roof plate the presence of SCO-spondin and slit2 in chick and *Xenopus leavis* embryos respectively promote fasciculation and drive the axons to the midline (Stanic et al., 2010; Tosa et al., 2015). Finally, at the midline, axons either turn to the ipsilateral side or continue to the opposite side. Worth noting, a set of EphA7-expressing cells at the midline seems critical in the axonal decision process (Stanic et al., 2014). The relevance of the caudal diencephalic roof plate in guiding the PC axons is also sustained by data from various null mutant mice (mutant for *pax2/5, pax6, msx 1*), which display a wide range of abnormalities in the diencephalic roof plate and fail to form a normal PC (Schwarz et al., 1999; Louvi and Wassef, 2000; Estivill-Torrus et al., 2001; Fernandez-Llebrez et al., 2004; Ramos et al., 2004).

**Figure 1:**
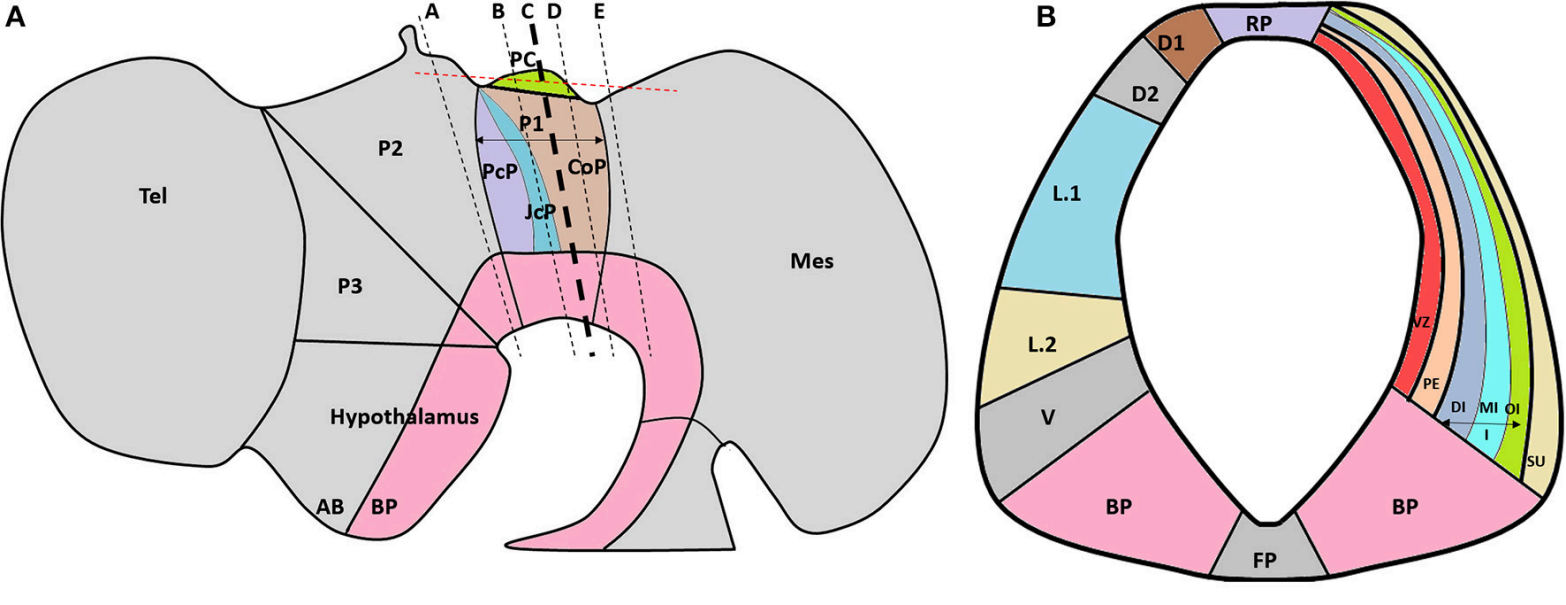
**(A)** Schematic lateral view of the HH29 chick brain, emphasizing with colors prosomere 1 subdivisions (PcP, JcP, and CoP) and PC localization in the CoP roof plate. The dotted black lines show the plane of the frontal sections (Figures 2, 4), and the red line shows the plane of the horizontal sections. **(B)** Schematic subdivision of the CoP from prosomere 1 showing the ventro-dorsal subdivision (left) and lateral subdivisions (right). A, alar plate; BP, basal plate; CoP, commissural pretectum; D1, dorsal 1 domain; D2, dorsal 2 domain; DI, deep layer from the intermediate stratum; FP, floor plate; JcP, juxtacommissural pretectum; I, intermediate stratum; L.1, lateral 1 subdomain; L.2, lateral 2 subdomain; Mes, mesencephalon; MI, medial layer from the intermediate stratum; OI, outer layer from the intermediate stratum; P1, prosomere 1; P2, prosomere 2; P3, prosomere 3; PC, posterior commissure; PcP, precommissural pretectum; PE, periventricular stratum; RP, roof plate subdomain; Tel, telencephalon; V, ventral subdomain; VZ, ventricular zone; Su, superficial stratum (Modified from Ferran et al., 2007, 2009).

Despite the important influence that extracellular molecules have on axonal guidance, there are few reports about the expression pattern of these molecules during CNS development. Furthermore, and to the best of our knowledge, there are no previous reports about ECM molecules during PC formation. Therefore, the aim of this work was to determine the expression patterns of seven ECM molecules (i.e., tenascin, human natural killer-1 [HNK-1], laminin, fibronectin, perlecan, decorin, and osteopontin) in relation to PC axons and SCO-spondin, a protein exclusively expressed in the roof plate of prosomere 1. The localizations of these molecules were established according to the genoarchitectural subdivisions reported by Ferran et al. (2007, 2009), who described ventro-dorsal (i.e., BP, V, L.2, L.1 D2, D.1, and RP) and lateral (i.e., VZ, PE, DI, MI, OI, SU) subdivisions of this prosomere (Figure 1).

## Materials and methods

### Chick embryos

Fertilized chick eggs were incubated at 38°C in a humidified incubator until HH24 or HH29 stage. Embryos were staged according to Hamburger and Hamilton (HH stage) (Hamburger and Hamilton, 1992). All animals were handled in strict accordance with Animal Welfare Assurance protocol and following the guidelines outlined in the Biosafety and Bioethics Manual of the National Commission of Scientific and Technological Research (CONICYT, Chilean Government). Additionally, all work with animals was approved by the Ethics and Animal Care and Use Committee of the University of Concepción, Chile.

### Immunohistochemistry

HH29 chick embryos were fixed for 24 h in Carnoy's solution, dehydrated in ascending alcohol concentrations, and embedded in paraplast. Brains were oriented to obtain frontal or horizontal sections (5–7 μm sections) of the prosomere 1, prosomere 2, or mesencephalon. Sections were immunostained with either a rabbit anti-neural cell adhesion molecule (NCAM) (AB5032, EMD Millipore) or a rabbit anti-Reissner's fiber glycoprotein antibody that recognizes SCO-spondin (kindly donated by E. Rodriguez; Caprile et al., 2009), as well as with one of the following anti-mouse ECM molecules (all obtained from the Developmental Studies Hybridoma Bank, University of Iowa): anti-laminin1 (3H11), anti-fibronectin (B3D6), anti-HNK-1 epitope (1C10), anti-perlecan (5C9), anti-decorin (CB1), anti-tenascin-C (M1B4), or anti-osteopontin (MPIIIB10). These antibodies were diluted in a Tris-HCl buffer containing 1% bovine serum albumin. As secondary antibodies, goat anti-mouse Alexa-546 and anti-rabbit Alexa-488 antibodies (Invitrogen) were diluted to 1:100 in a Tris-HCl buffer containing 1% bovine serum albumin and incubated for 2 h at room temperature. Nuclei were visualized with TO-PRO-3 staining (Invitrogen). Images were acquired with a spectral confocal Zeiss LSM780 microscope.

## Results

The PC is located at the dorsocaudal border of the prosomere 1 commissural domain (Puelles and Rubenstein, 2003; Ferran et al., 2007, 2009; Figure 1). This commissure is formed by ventral and dorsal pretectal neuron axons that run dorsally to the midline (Ware and Schubert, 2011). The pioneer PC axons have been reported at HH18-HH19, although until HH24 the PC is not well conformed (Schoebitz et al., 1986; Didier et al., 1992, 2007; Caprile et al., 2009). As a first approach to analyze the ECM during PC development we perform immunohistochemistry at HH24 (Supplementary Figure 1) founding that at this stage laminin, HNK-1, and osteopontin are expressed in the prosomere 1, in contrast with tenascin and fibronectin that are not detected. At this stage the detected ECM molecules display a diffuse expression pattern, that is not specially related with the axons that begin to conform the PC.

**Figure 2:**
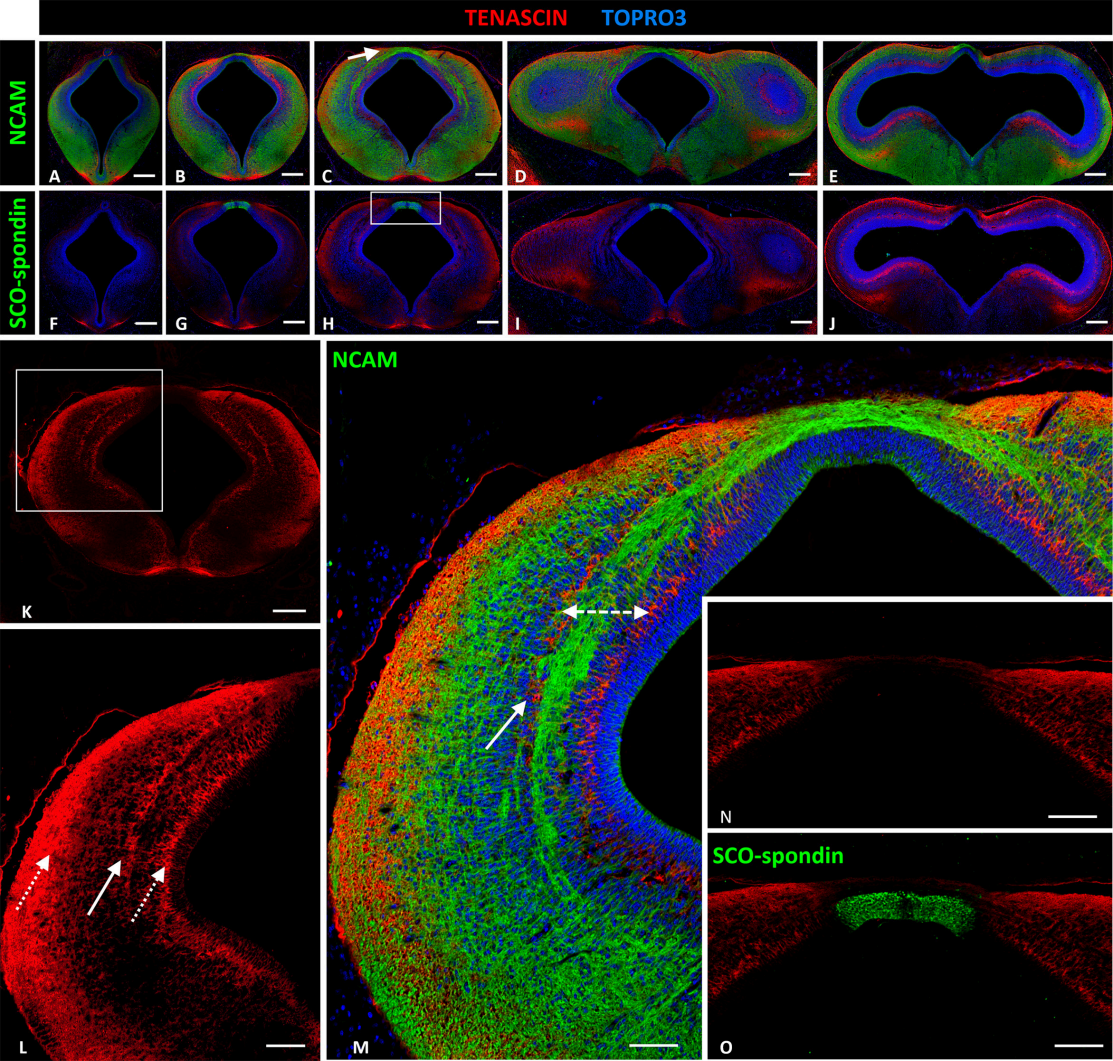
**Expression of tenascin, SCO-spondin, and NCAM in frontal sections of HH29 chick diencephalon and mesencephalon. (A–E)** Confocal images showing the co-localization of NCAM (green) and tenascin (red) in the region between prosomere 2 and the mesencephalon. The PC is located in the medial region of prosomere 1 (arrow in **C**). **(F–J)** Confocal images showing co-localization of SCO-spondin (green) and tenascin (red) in frontal sections between prosomere 2 and the mesencephalon, showing the presence of SCO-spondin only in the roof plate of prosomere 1 **(G–I)**. **(K–M)** Higher magnification of **(C)** showing tenascin arrangement into three columns (arrows in **L**). The axonal fascicles of the PC extended inside a corridor (dotted double-arrow in **M**) delimited by the tenascin columns. **(N,O)** Higher magnification of the area framed in **(H)** showing the absence of tenascin in the roof plate, coinciding with the expression of SCO-spondin. TOPRO 3 (blue) was used as nuclear counterstain. Scale bars = 200 μm in **(A–K)**; 100 μm in **(L)**; and 50 μm in **(M–O)**.

At HH29 the PC is well developed and the prosomere1 has been highly characterized at anatomical level (Ferran et al., 2009). To define the study area, anti-NCAM immunohistochemistry staining was performed in the area between prosomere 2 (Figure 2A) and the most cephalic region of the mesencephalon of HH29 chick embryos (Figure 2E). The location of the fibers was in accordance with the ventro-dorsal columns reported by Ferran et al. (2009, Figure 4), and although we do not perform analysis of Pax6, the width of these columns allowed us to locate the PC axonal tract in the deep layer of the intermediate stratum of prosomere 1, especially in the medial (Figure 2C) and caudal (Figure 2D) regions (arrow in Figure 2C). However, there may be also some fibers in the outer half of the periventricular stratum (Figures 2B–D). SCO-spondin expression was also analyzed since this protein is specifically expressed at the prosomere 1 roof plate (Figures 2G–I), serving as an anatomical indicator, and since it has been related to PC axon fasciculation.

### Extracellular matrix proteins present a specific expression pattern in the medial zone of prosomere 1 of HH29 chick embryos

#### Tenascin and HNK-1

Tenascin expression in the region between prosomere 2 and the mesencephalon was arranged into two ventro-dorsal columns extending from the basal plate to the dorsal region (D1). One column was located in the superficial stratum, while the other was located in the periventricular stratum (Figure 2K, and dotted arrows Figure 2L). Tenascin was absent in the roof and basal plates (Figures 2F–J). In prosomere 1, especially in the medial region, a third column appeared from the medial layer of the intermediate stratum (solid arrow Figures 2L,M). This third column, in conjunction with the periventricular column, formed a corridor that surrounded the axon fascicles located at the intermediate stratum (dotted double arrow Figure 2M). Tenascin expression abruptly decreased in the dorsal region (D.1) (Figure 2N) and the roof plate, coinciding with SCO-spondin expression (Figure 2O). In the basal region, this protein was present in the floor plate of prosomeres 1 and 2.

**Figure 3:**
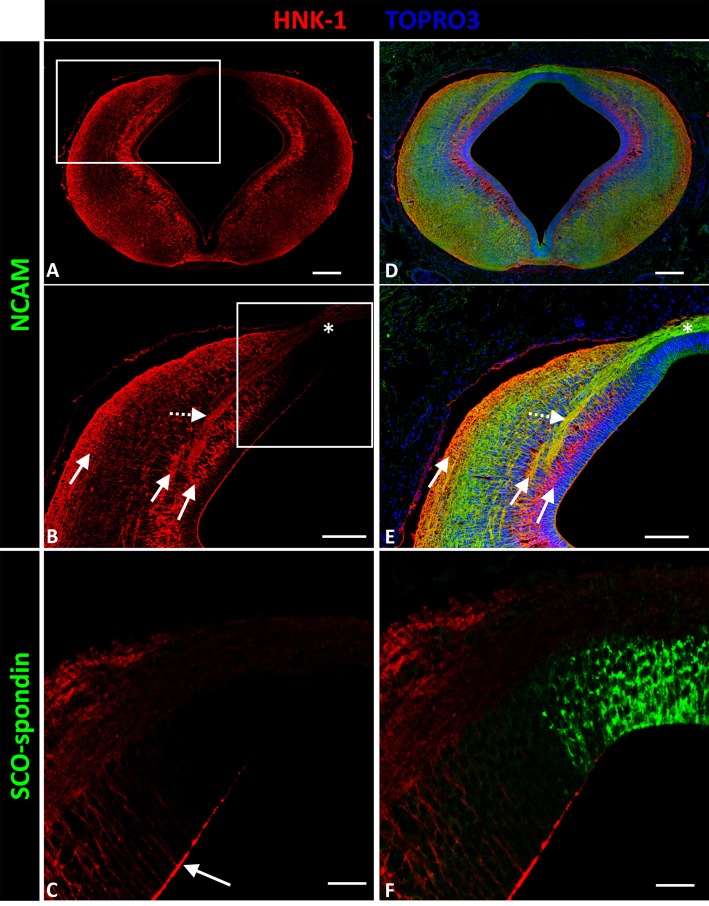
**Expression of HNK-1, SCO-spondin, and NCAM in frontal sections of chicken prosomere 1 at stage HH29. (A,B,D,E)** Confocal images showing co-localization of NCAM (green) and HNK-1(red). HNK-1 is arranged into three columns (solid arrows in **B**). The axonal fascicles of the PC extended inside a corridor formed by these columns. The axons of the PC are immunopositive for HNK-1 in the lateral regions (dotted arrow in **E**), but the immunoreactivity highly diminishes in the dorsal region and in the roof plate (asterisk in **B,E**). **(C,F)** Confocal images showing co-localization of SCO-spondin (green) and NHK-1(red) in an area equivalent to the area framed in **(B)**, showing the absence of HNK-1 in the roof plate, coinciding with the expression of SCO-spondin. HNK-1 is present in the apical region of the neuroepithelial cells (arrow in **C**), with the exception of roof plate cells positive for SCO-spondin. TOPRO 3 (blue) was used as nuclear counterstain. Scale bars = 100 μm in **(A,B,D,E)**; 25 μm in **(C,F)**.

HNK-1 expression was almost identical to tenascin expression (Figures 3A,D), with three ventro-dorsal columns extending from the basal plate to D.1 and located in the periventricular, medial intermediate, and superficial strata (solid arrows in Figures 3B,E). The first two columns delimited axon fascicles extending into the dorsal region. Additionally, HNK-1 co-localized with axons in the intermediate stratum of L.1 (dotted arrow in Figures 3B,E), but this co-localization disappeared as axons arrived at the roof plate (asterisk in Figures 3B,E). HNK-1 was also found in the apical region of neuroepithelial cells (arrow in Figure 3C), with the exception of roof plate cells secreting SCO-spondin (Figure 3F).

#### Osteopontin

**Figure 4:**
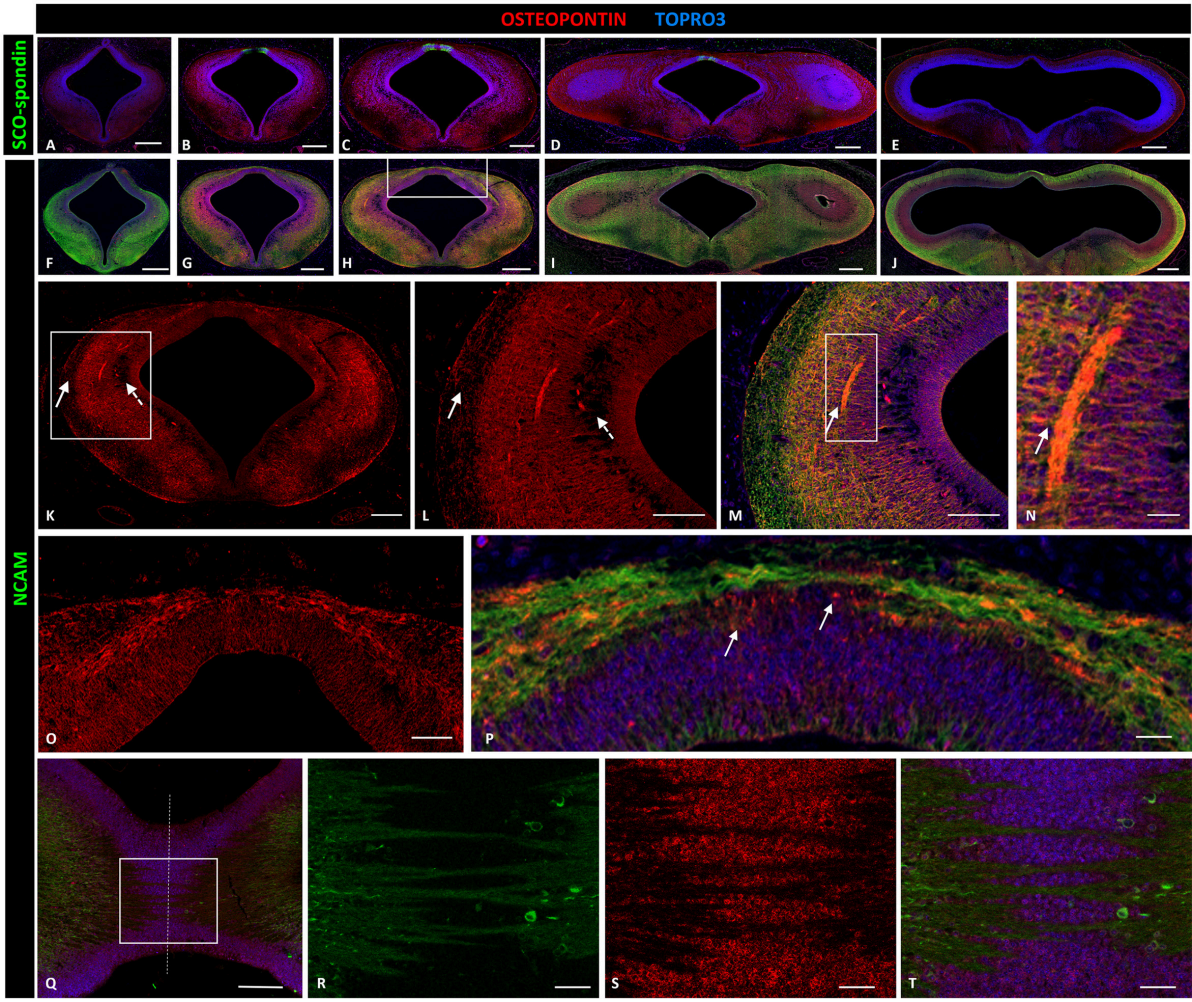
**Expression of osteopontin, SCO-spondin, and NCAM in frontal and horizontal sections of chicken diencephalon and mesencephalon at stage HH29**. Double immunohistochemistry with anti-SCO-spondin (**A**–**E**, green) or NCAM (**F–J**, green) and osteopontin (red) in the region between prosomere 2 and mesencephalon. Osteopontin was highly expressed in prosomere 1 **(B–D)** but not in prosomere 2 **(A)** or the mesencephalon **(E)**. **(K)** Expression of osteopontin in the middle region of prosomere 1. **(L–N)** Magnification of the region framed in **(K)** showing the wide expression of osteopontin, with the exception of the periventricular and superficial strata (dotted and continuous arrows, respectively, in **K,L**). **(N)** Higher magnification of the area framed in **(M)** showing the co-localization of NCAM and osteopontin in the axonal fascicle. **(O,P)** Magnification of the area framed in **(H)** showing osteopontin expression in the dorsal region and in the roof plate, between the PC axons. **(Q–T)** Horizontal sections of the roof plate of prosomere 1 showing the axons crossing the midline (dotted line) and immersed in a osteopontin-rich medium. **(R–T)** magnification of the area framed in **(Q)**. TOPRO 3 (blue) was used as nuclear counterstain. Scale bars = 200 μm in **(A–J)**; 100 μm in **(K)**; 50 μm in **(L,M,O,Q)**; 10 μm in **(N,P,R,S)**.

Osteopontin was highly expressed in the entire prosomere 1 region (Figures 4B–D), in contrast to the cephalic (prosomere 2, Figure 4A) and caudal (mesencephalon, Figure 4E) limits where osteopontin expression drastically decreased. In prosomere 1, osteopontin was found along the intermediate stratum but not in the superficial or periventricular strata (continuous and dotted arrows, respectively, in Figures 4K,L). Osteopontin expression increased along the axon trajectory, and both axons and osteopontin co-localized in the lateral region (arrows in Figures 4M,N). In the dorsal roof plate, osteopontin was found between the axons traversing the medial region. Here, the diencephalic roof plate cells were immunopositive for osteopontin, suggesting possible osteopontin secretion by these cells (arrows in Figure 4P). The horizontal sections along the roof plate presented osteopontin between the axons traversing the midline (Figures 4Q–T).

#### Laminin

**Figure 5:**
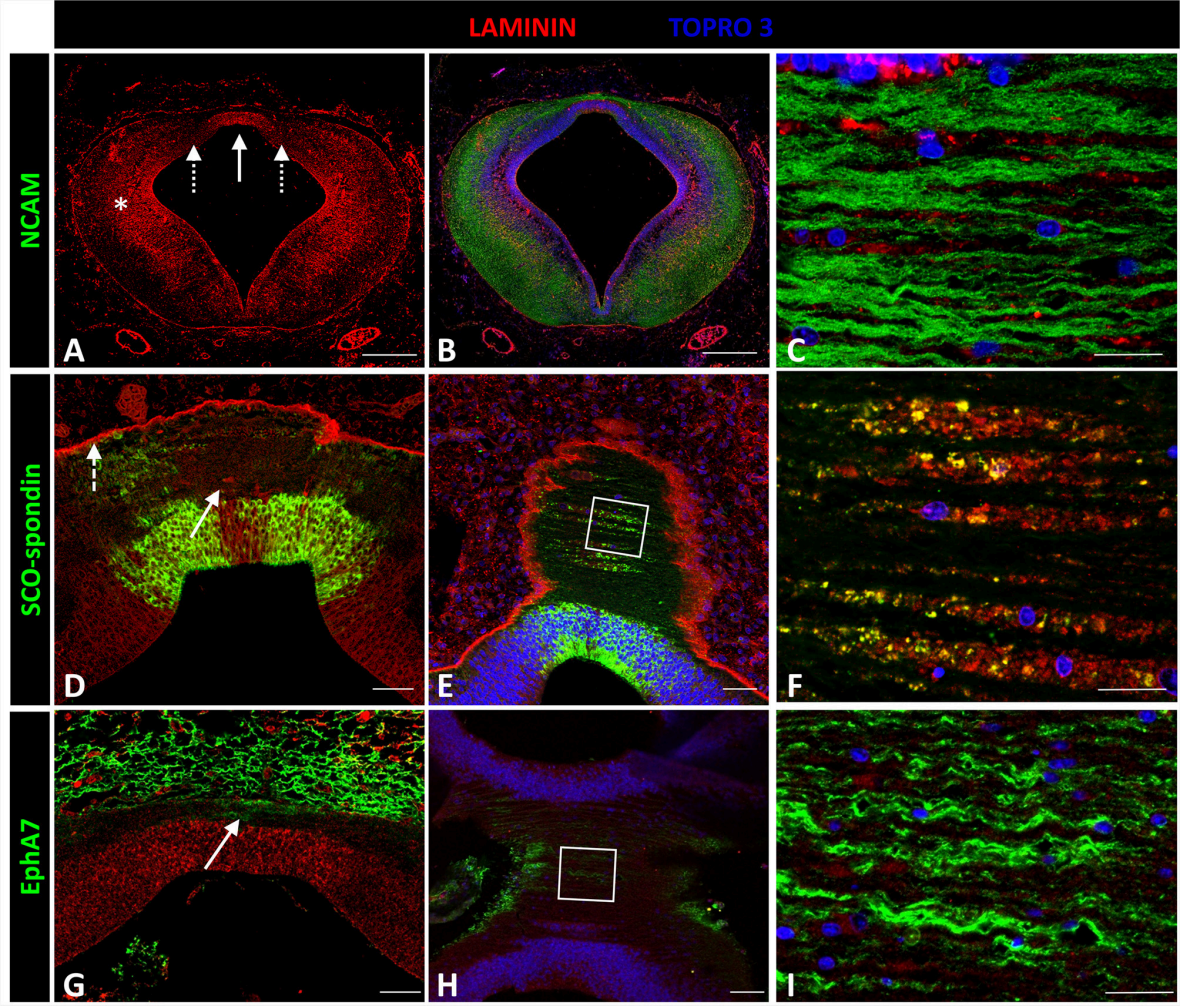
**Expression of laminin, SCO-spondin, EphA7, and NCAM in frontal and horizontal sections of chicken prosomere 1 at stage HH29. (A–C)** Double immunohistochemistry with anti-NCAM (green) and laminin (red) showing the broad expression of this ECM molecule, especially in the lateral region (asterisk in **A**) and in the roof plate (solid arrow in **A**). **(C)** Coronal section of the roof plate reveals the presence of laminin in the ECM surrounding the axons. **(D)** Frontal section of the roof plate showing the presence of laminin in the midline (solid arrow) and in the external limiting membrane (dotted arrow). **(E,F)** Horizontal images of the prosomere 1 roof plate showing that laminin colocalizes with SCO-spondin in the ECM surrounding the axons. **(F)** Magnification of the area framed in **(E)**. **(G–I)** Double immunohistochemistry with anti-EphA7 (green) and laminin (red) showing the expression of EphA7 in the dorsal midline cells (arrow in **G**). **(H,I)** Horizontal images of the prosomere 1 roof plate showing that laminin is in close contact with the basal prolongations of the midline cells positives for EphA7. **(I)** Magnification of the area framed in **(H)**. TOPRO 3 (blue) was used as nuclear counterstain. Scale bars = 200 μm in **(A,B)**; 20 μm in **(D,E)**; 10 μm in **(C,F)**.

Laminin expression in the prosomere 1 medial region was recorded from the basal plate to the roof plate, with maximal expressions in the lateral region (asterisk in Figures 5A,B) and roof plate (solid arrow in Figures 5A,D), and minor expression in the dorsal region (dotted arrows in Figure 5A). To more fully analyze expression in the roof plate, horizontal sectioning of this region was conducted (Figures 5C,E,F). Through this, laminin was found located in the ECM surrounding the axons (Figure 5C), co-localizing with SCO-spondin (Figure 5F). Laminin was also highly expressed in the external limiting membrane (dotted arrow in Figure 5D). The expression of laminin was also analyzed in relation with EphA7, a transmembrane protein located in the basal prolongations of the dorsal midline cells that traverse the PC (arrow in Figure 5G). Horizontal sections showed the presence of EphA7 in close contact with the laminin of the extracellular space (Figure 5I).

#### Fibronectin

**Figure 6:**
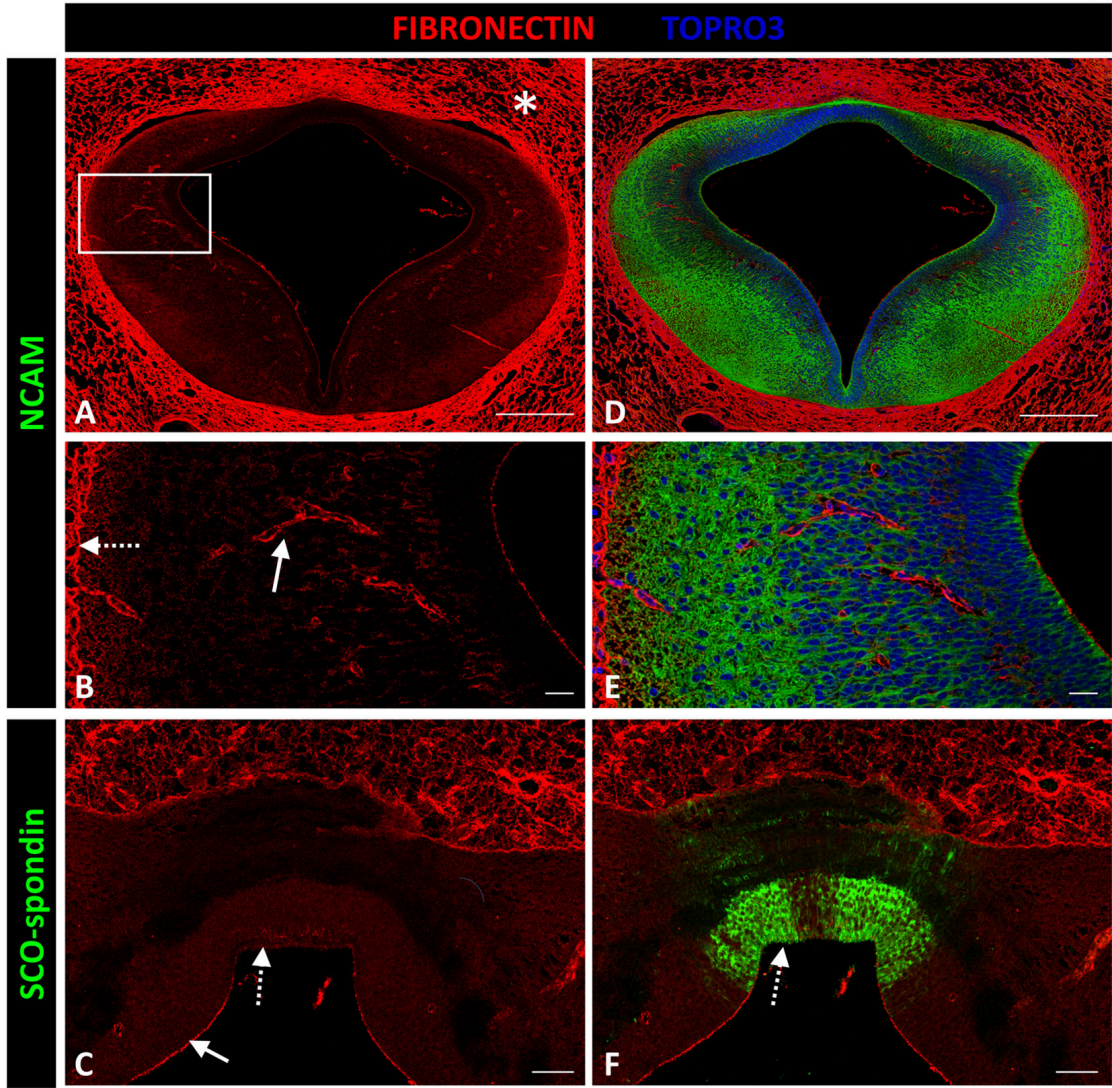
**Expression of fibronectin, SCO-spondin, and NCAM in frontal sections of chicken prosomere 1 at stage HH29. (A–D)** Complete section of prosomere 1. **(B–E)** Magnification of the area framed in **(A)**. **(C–F)** Dorsal and roof plate of prosomere 1. **(A–C)** Confocal images showing the localization of fibronectin (red) in the mesenchyme that surrounds the CNS (asterisk in **A**), in the blood vessel walls (solid arrow in **B**), in the external limiting membrane (dotted arrow in **B**), and in the apical membrane of neuroepithelial cells (solid arrow in **C**), except for the cells forming the roof plate (dotted arrow in **C**), which, in contrast, were positive for SCO-spondin (arrow in **F**). **(D–F)** Same sections as in **(A–C)**, respectively, immunostained with **(D,E)** NCAM or **(F)** SCO-spondin. TOPRO 3 (blue) was used as nuclear counterstain. Scale bars = 200 μm in **(A,D)**; 20 μm in **(B,C,E,F)**.

Fibronectin expression was not detected in the prosomere 1 medial region except in blood vessel walls (solid arrow in Figure 6B), the external limiting membrane (dotted arrow in Figure 6B), and the apical region of neuroepithelial cells (arrow in Figure 6C), with the exception of cells expressing SCO-spondin at the roof plate cells (dotted arrow in Figures 6C–F). However, fibronectin was highly expressed in mesenchymal tissue surrounding the CNS (asterisk in Figure 6A).

#### Decorin and perlecan

**Figure 7:**
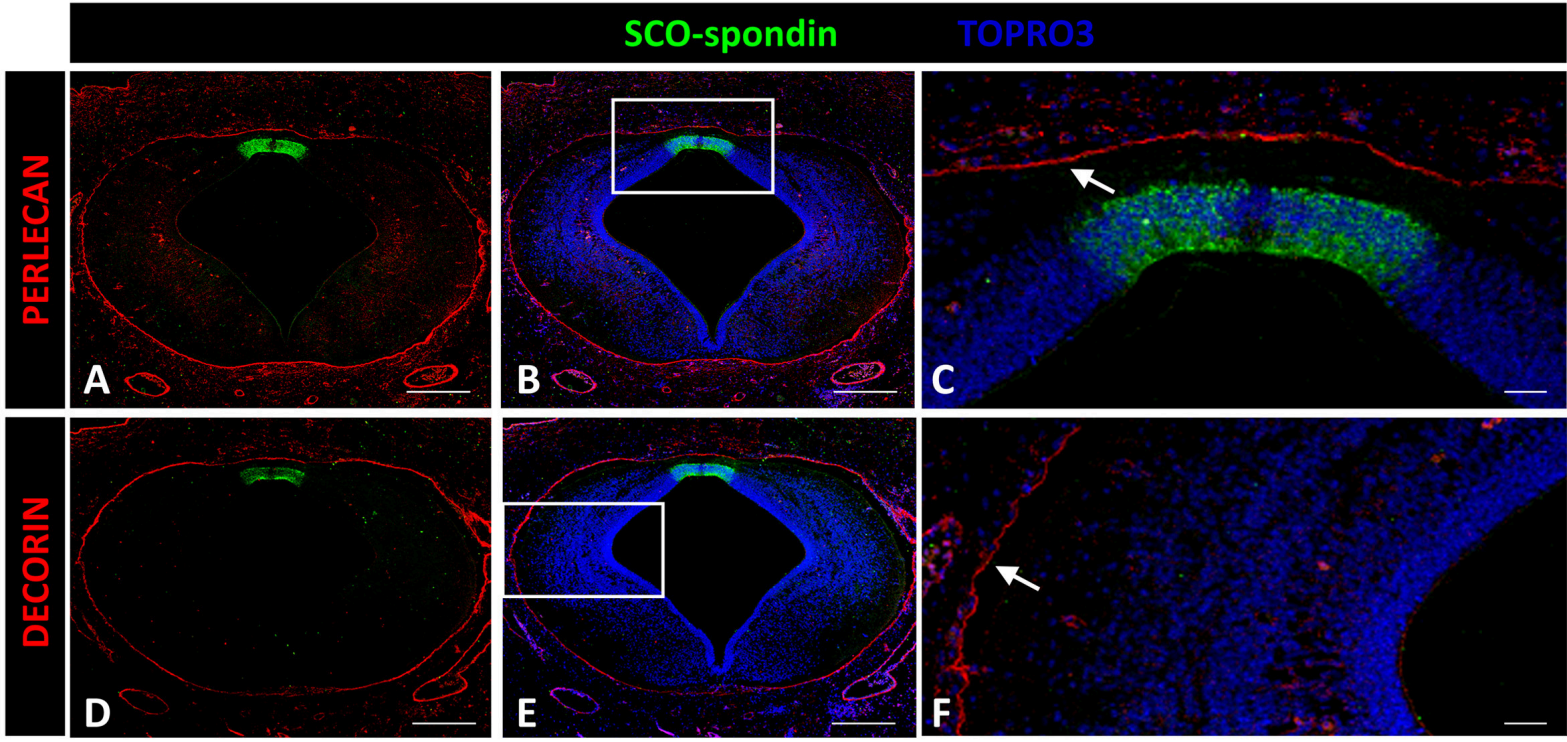
**Expression of decorin and perlecan in chicken prosomere 1 at stage HH29**. Immunohistochemistry against perlecan **(A–C)** or decorin **(D,E)** and SCO-spondin (green). **(C,F)** Higher magnification of the areas framed in **(B)** and **(E)**, respectively, showing the absence of both molecules in the roof plate and in the lateral region. The localization of perlecan and decorin is restricted to the external limiting membrane (arrows in **C,F**). TOPRO 3 was used as nuclear counterstain. Scale bars = 200 μm in **(A,B,D,E)**; 25 μm in **(C,F)**.

Decorin and perlecan expressions were both circumscribed to the external limiting membrane and to blood vessel walls (Figure 7).

## Discussion

The present work reports the expression patterns of different ECM molecules during PC development. In other contexts, these molecules have been involved in axonal guidance, and the specific expressions observed in this study suggest a possible role of these components in correct PC development.

**Figure 8:**
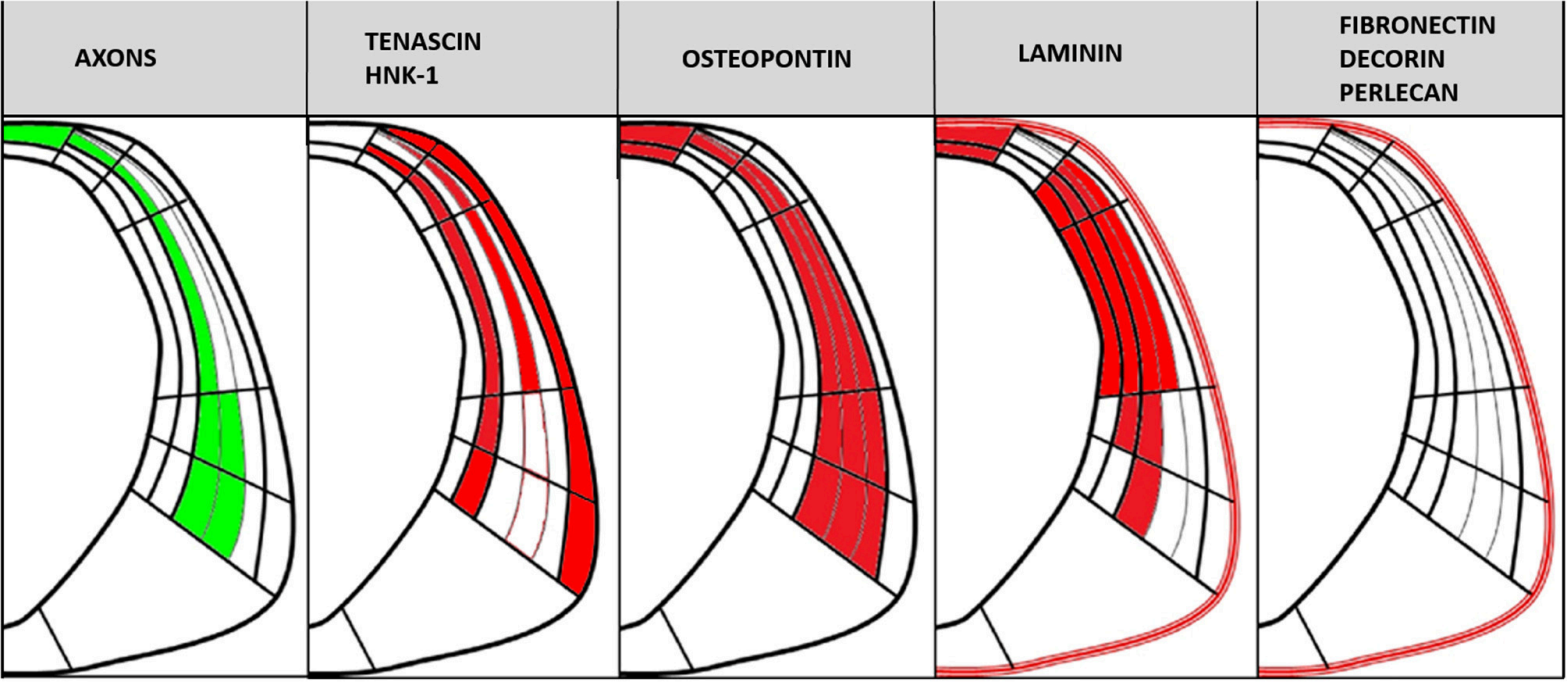
**Scheme showing PC axonal trajectory and the localization of the PC axons and ECM molecules analyzed in this study in relation to the CoP subdivisions detailed in Figure 1B**.

Confocal images of HH29 embryos showed that PC axons were embedded in a potentially rich molecular ECM environment containing different molecules with specific expression patterns (Figure 8). These molecules were classified into the following three groups based on axonal trajectory: (a) molecules that formed barriers delimiting axon fascicle trajectory, such as tenascin and HNK1; (b) molecules located very close to axons, especially in the roof plate, such as osteopontin and laminin; and (c) molecules unrelated to axonal trajectory, such as fibronectin, decorin, and perlecan.

### ECM molecules that delimit axonal trajectory

#### Tenascin

The tenascin (TN) gene family includes distinct genes (i.e., TN-C, R, X, and Y), but only TN-C and TN-R have been reported in CNS tissues (Faissner, 1997). Both proteins are characterized by a serial arrangement of a cysteine-rich amino-terminus and varying numbers of fibronectin-type and EGF-type domains (Faissner, 1997). The antibody used in the present study recognize TN-C (Akbareiana et al., 2013).

TN-C has been related to various aspects of CNS development, including the regulation of proliferation and differentiation processes, participation in axon guidance, and the modulation of responsiveness to other cell matrix components, such as FGF2 (Gotz et al., 1997; Besser et al., 2012). Furthermore, the importance of TN during commissure development was studied in the optic tract. Specifically, TN was found bordering the developing optic projection, thus preventing optic axons from exiting the optic tract in a medio-caudal direction. TN inhibition in this context results in axon misrouting and increased axonal branches (Becker et al., 2003). The TN expression pattern found in the present study suggests a similar function during PC development. In particular, tenascin was located at the border of dorsally extending axonal fascicles, and this positioning could generate a repulsion barrier that would prevent axons from leaving the fascicle.

#### HNK-1

The HNK-1 carbohydrate epitope is comprised of a unique trisaccharide, HSO3-3GlcAb1-3Galb1-4GlcNAc. This epitope is only carried by certain molecule types, such as cell adhesion molecules (e.g., NCAM, L1; Ong et al., 2002) or some ECM proteins, especially tenascin (Yagi et al., 2010). This glycoepitope is predominantly expressed in the nervous system, being used as axonal marker in some anamniotes (Ware et al., 2015), and contributes to regulating neural functions (Senn et al., 2002; Yamamoto et al., 2002; Kizuka et al., 2006; Morita et al., 2009).

The observed localization of HNK-1 during PC development suggests that it was carried by tenascin, particularly as both molecules presented similar expression patterns consisting of three ventro-dorsal columns. Additionally, HNK-1 was present in L.1 and D.2 located axons, but expression decreased as the axons reached the dorsal region and was absent in the roof plate region.

These results suggest the participation of HNK-1 and tenascin in guiding axons from the ventral to the dorsal region of the prosomere 1 through the generation of an axonal corridor limited by repulsive tenascin and HNK-1 walls.

### ECM molecules in close contact with PC axons

#### Laminin

Laminins are heterotrimeric glycoproteins consisting of α, β, and γ subunits that assemble into a characteristic cruciform structure. In turn, this structure is a major ECM component during the development and in the mature CNS. Currently, 11 laminin chains have been identified, including α1–5, β1–3, and γ1–3. These chains combine to form up to 15 different laminin isoforms (for a review see Colognato and Yurchenco, 2000). Laminins play important roles *in vitro* in migration, differentiation, and axonal growth for a variety of neuronal subtypes (Liesi et al., 1989; Adams et al., 2005; Turney and Bridgman, 2005).

The antibody used in the present study recognize laminin 1. In culture, when laminin 1 is used as substratum, it promotes robust axon outgrowth for a variety of CNS and peripheral nervous system neurons. Analyses of laminin mutants in *Caenorhabditis elegans, Drosophila, Danio rerio*, and *Mus musculus* provide strong evidence that this protein is required for correct axon guidance (Garcia-Alonso et al., 1996; Karlstrom et al., 1996; Forrester and Garriga, 1997; Huang et al., 2003; Paulus and Halloran, 2006; Chen et al., 2009). Indeed, *bashful*/Laminin-α1 zebrafish mutants present defects in most CNS axon pathways, including the PC (Paulus and Halloran, 2006). Additionally, axon tracts in *bashful* mutants display defects in fasciculation and extension, as well as increased branching. Interestingly, and in contrast to CNS axons, most peripheral axons appear normal in *bashful* mutants. In mice, the conditional knockout of laminin γ1 subunit in cortical neurons results in profound defects in neuronal migration and morphogenesis (Chen et al., 2009).

A number of *in vitro* studies demonstrate that laminin1 not only acts as a simple permissive substratum but, if provided locally, can also direct growing axons (Adams et al., 2005; Turney and Bridgman, 2005). Laminin isoforms primarily stimulate axon extension through adhesion and by signaling downstream of integrin receptors (Kuhn et al., 1995). Nevertheless, other receptors, such as syndecans or dystroglycan, may be involved in certain neurons (Hamill et al., 2009). Regarding the PC, integrin β1 has been found in PC axons (Caprile et al., 2009) and could therefore be a possible receptor for the laminin found in the present analyses.

Although, laminin alone has potent effects on neuronal morphogenesis, this protein could also function by modulating responses to axon guidance cues, such as netrins (Hopker et al., 1999) and different Eph/ephrin family members. In this context, while EphB displays no effect on retinal ganglion cell axons when growing on the cell adhesion molecule L1, when cultured on a combined laminin-L1 substratum, growth cones pause but remain dynamic in response to EphB (Suh et al., 2004). Laminin-mediated modulation can be important for PC development since Eph family members have been reported at the midline, where axons must decide to cross or not cross the midline (Stanic et al., 2014). At this point, PC axons (positive for NCAM, similar to L1) become defasciculated, and the presence of laminin may be important to pause the growth cones, thereby allowing the axons to detect Eph members expressed at the midline. The presence of laminin can also switch or nullify inhibitory axon guidance cues. For example, ephrin-A5 is an important repulsive factor toward retinal ganglion cell axons. This repulsive effect is maintained *in vitro* by retinal neurons using fibronectin as a substrate. However, when cultured on laminin, retinal ganglion cells are attracted toward a gradient of soluble ephrin-A5 (Weinl et al., 2003).

Considering the modulatory effects of laminin on Eph/ephrin family members, detailed analyses were performed to establish the presence of this protein in the roof plate, where EphA7 (Figures 5G–I; Stanic et al., 2014) and other Eph/ephrin family members have been found (results not published). In the roof plate, laminin was located in the ECM co-localizing with SCO-spondin and in close contact with PC axons and basal prolongations of the roof plate cells, positives for EphA7. The PC axons expressed integrin β1, a potential receptor for laminin and SCO-spondin, thus suggesting that laminin might modulate the effects of both SCO-spondin and the EphA7.

#### Osteopontin

At postnatal stage, osteopontin is principally associated with peripheral axon regeneration and it is upregulated during CNS injury. However, osteopontin appears to have different roles depending on axon type. For example, this protein increases motor axon outgrowth (Wright et al., 2014) but inhibits sensory axon outgrowth (Kury et al., 2005). Furthermore, postnatal hippocampal neurons cultured in an osteopontin substrate show more primary neurites than neurons grown in a laminin substrate (Plantman, 2012). Osteopontin expression and function during CNS development has not been previously described, but *in vitro* experiments with embryonic retinal ganglion cell neurons show that using this protein as a substrate promotes axonal growth, and the additional presence of laminin produces an additive effect (Ries et al., 2007).

Osteopontin contains an Arg-Gly-Asp cell-binding sequence specific to integrins. In fact, it has been reported the binding to αvβ3, αvβ1, αvβ5, and α9β1, and α4β1 integrin (Liaw et al., 1995; Smith et al., 1996; Bayless et al., 1998). PC axons express the β1 integrin subunit, although the alpha subunit has not been described, suggesting a possible axon-osteopontin interaction through these receptors.

### ECM molecules unrelated to axonal trajectory

#### Fibronectin

Fibronectin has been broadly used *in vitro* as a substrate for embryonic neurons due to its permissive effect on axonal growth. However, fibronectin expression in embryonic mice is restricted to the pial surface and blood vessel walls, with expression absent in the CNS (Milner and Campbell, 2002; Lathia et al., 2007). The presently obtained results revealed the same expression pattern in chick embryos, with expression restricted to the external membrane and blood vessels. Therefore, fibronectin does not appear to participate in the guidance of commissural axons.

#### Perlecan

Perlecan is a major heparan sulfate proteoglycan constituent of basement membranes. Additionally, perlecan surrounds axonal fascicle bundles from olfactory sensory neurons in embryonic mice (Shay et al., 2008). Perlecan can also promote neurite extension *in vitro* (Nakamura et al., 2015). In chick embryos, the expression pattern of this protein has been studied until HH17, where, as in the present report, perlecan becomes circumscribed to the external limiting membrane (Soulintzi and Zagris, 2007) and does not appear to participate in axon guidance.

#### Decorin

Previous studies have been unable to detect decorin expression in mouse brain embryos, with the exception of the meninges and a region of the fourth ventricle floor plate (Scholzen et al., 1994). The present work found a similar expression pattern in chick embryos, with expression restricted to the external limiting membrane.

In conclusion, the present results support that posterior commissure axons navigate a complex extracellular matrix. While some molecules in this matrix generate a barrier surrounding the axons, others are expressed in the roof plate. At the midline, axons must decide whether or not to cross to the contralateral side in a medium containing laminin, osteopontin, SCO-spondin, and members of the Eph/ephrin family. Understanding the interrelation and modulation of these molecules in axonal guidance will provide a clearer image of how each axon navigates in a highly specific manner to find its synaptic counterpart. This knowledge will also aid in the creation of new regenerative therapies for CNS injuries, a field in which extracellular matrix technology is particularly promising (Ren et al., 2015).

## Author contributions

KS and NS carried out most of the experiments, performed the immunohistochemistry, embryo fixation, and dissection. BF contributed to the immunohistochemistry experiments. MT and HM contributed intellectually to experimental design and interpretation, and contributed to writing the manuscript. TC directed the project, designed and interpreted the experiments and wrote the manuscript.

## Funding

This research was supported by FONDECYT 1110723 and Enlace UdeC 216.031.112-1.0 awarder to TC and FONDECYT1140394 awarded to MT.

### Conflict of interest statement

The authors declare that the research was conducted in the absence of any commercial or financial relationships that could be construed as a potential conflict of interest.

## References

[B1] Adams D. N. Kao E. Y. Hypolite C. L. Distefano M. D. Hu W. S. Letourneau P. C. (2005). Growth cones turn and migrate up an immobilized gradient of the laminin IKVAV peptide. J. Neurobiol. 62, 134–147. 10.1002/neu.20075 15452851

[B2] Akbareiana S. Nagyb N. Steigera C. E. Mablyc J. D. Millera S. A. Hottaa R.. (2013). Enteric neural crest-derived cells promote their migration by modifying their microenvironment through tenascin-C production. Dev. Biol. 382, 446–456. 10.1016/j.ydbio.2013.08.006 23958436 PMC3800188

[B3] Barros C. S. Franco S. J. Muller U. (2011). Extracellular matrix: functions in the nervous system. Cold Spring Harb. Perspect. Biol. 3:a005108. 10.1101/cshperspect.a005108 21123393 PMC3003458

[B4] Bayless K. J. Meininger G. A. Scholtz J. M. Davis G. E. (1998). Osteopontin is a ligand for the alpha4beta1 integrin. J. Cell Sci. 111, 1165–11174. 9547293 10.1242/jcs.111.9.1165

[B5] Becker C. G. Schweitzer J. Feldner J. Becker T. Schachner M. (2003). Tenascin-R as a repellent guidance molecule for developing optic axons in zebrafish. J. Neurosci. 23, 6232–6237. 12867507 10.1523/JNEUROSCI.23-15-06232.2003 PMC6740549

[B6] Besser M. Jagatheaswaran M. Reinhard J. Schaffelke P. Faissner A. (2012). Tenascin C regulates proliferation and differentiation processes during embryonic retinogenesis and modulates the de-differentiation capacity of Muller glia by influencing growth factor responsiveness and the extracellular matrix compartment. Dev. Biol. 369, 163–176. 10.1016/j.ydbio.2012.05.020 22691363

[B7] Caprile T. Osorio G. Henriquez J. P. Montecinos H. (2009). Polarized expression of integrin beta1 in diencephalic roof plate during chick development, a possible receptor for SCO-spondin. Dev. Dyn. 238, 2494–2504. 10.1002/dvdy.22070 19681158

[B8] Chen Z. L. Haegeli V. Yu H. Strickland S. (2009). Cortical deficiency of laminin gamma1 impairs the AKT/GSK-3beta signaling pathway and leads to defects in neurite outgrowth and neuronal migration. Dev. Biol. 327, 158–168. 10.1016/j.ydbio.2008.12.006 19118544 PMC2669444

[B9] Chilton J. K. (2006). Molecular mechanisms of axon guidance. Dev. Biol. 292, 13–24. 10.1016/j.ydbio.2005.12.048 16476423

[B10] Colognato H. Yurchenco P. D. (2000). Form and function: the laminin family of heterotrimers. Dev. Dyn. 218, 213–234. 10.1002/(SICI)1097-0177(200006)218:2<213::AID-DVDY1>3.0.CO;2-R 10842354

[B11] Dickson B. J. Zou Y. (2010). Navigating intermediate targets: the nervous system midline. Cold Spring Harb. Perspect. Biol. 2:a002055. 10.1101/cshperspect.a002055 20534708 PMC2908764

[B12] Didier R. Meiniel R. Meiniel A. (1992). Monoclonal antibodies as probes for the analysis of the secretory ependymal differentiation in the subcommissural organ of the chick embryo. Dev. Neurosci. 14, 44–52. 10.1159/000111646 1600879

[B13] Didier R. Meiniel O. Meiniel A. (2007). Molecular cloning and early expression of chick embryo SCO-spondin. Cell Tissue Res. 327, 111–119. 10.1007/s00441-006-0259-4 16900377

[B14] Dityatev A. Seidenbecher C. I. Schachner M. (2010). Compartmentalization from the outside: the extracellular matrix and functional microdomains in the brain. Trends Neurosci. 33, 503–512. 10.1016/j.tins.2010.08.003 20832873

[B15] Estivill-Torrus G. Vitalis T. Fernandez-Llebrez P. Price D. J. (2001). The transcription factor Pax6 is required for development of the diencephalic dorsal midline secretory radial glia that form the subcommissural organ. Mech. Dev. 109, 215–224. 10.1016/S0925-4773(01)00527-5 11731235

[B16] Faissner A. (1997). The tenascin gene family in axon growth and guidance. Cell Tissue Res. 290, 331–341. 10.1007/s004410050938 9321695

[B17] Fernandez-Llebrez P. Grondona J. M. Perez J. Lopez-Aranda M. F. Estivill-Torrus G. Llebrez-Zayas P. F.. (2004). Msx1-deficient mice fail to form prosomere 1 derivatives, subcommissural organ, and posterior commissure and develop hydrocephalus. J. Neuropathol. Exp. Neurol. 63, 574–586. 10.1093/jnen/63.6.574 15217086

[B18] Ferran J. L. de Oliveira E. D. Merchan P. Sandoval J. E. Sanchez-Arrones L. Martinez-De-La-Torre M.. (2009). Genoarchitectonic profile of developing nuclear groups in the chicken pretectum. J. Comp. Neurol. 517, 405–451. 10.1002/cne.22115 19790262

[B19] Ferran J. L. Sanchez-Arrones L. Sandoval J. E. Puelles L. (2007). A model of early molecular regionalization in the chicken embryonic pretectum. J. Comp. Neurol. 505, 379–403. 10.1002/cne.21493 17912743

[B20] Forrester W. C. Garriga G. (1997). Genes necessary for *C. elegans* cell and growth cone migrations. Development 124, 1831–1843. 9165130 10.1242/dev.124.9.1831

[B21] Garcia-Alonso L. Fetter R. D. Goodman C. S. (1996). Genetic analysis of Laminin A in Drosophila: extracellular matrix containing laminin A is required for ocellar axon pathfinding. Development 122, 2611–2621. 8787736 10.1242/dev.122.9.2611

[B22] Gotz M. Bolz J. Joester A. Faissner A. (1997). Tenascin-C synthesis and influence on axonal growth during rat cortical development. Eur. J. Neurosci. 9, 496–506. 10.1111/j.1460-9568.1997.tb01627.x 9104592

[B23] Hamburger V. Hamilton H. L. (1992). A series of normal stages in the development of the chick embryo. 1951. Dev. Dyn. 195, 231–272. 10.1002/aja.1001950404 1304821

[B24] Hamill K. J. Kligys K. Hopkinson S. B. Jones J. C. (2009). Laminin deposition in the extracellular matrix: a complex picture emerges. J. Cell Sci. 122(Pt 24), 4409–4417. 10.1242/jcs.041095 19955338 PMC2787456

[B25] Hopker V. H. Shewan D. Tessier-Lavigne M. Poo M. Holt C. (1999). Growth-cone attraction to netrin-1 is converted to repulsion by laminin-1. Nature 401, 69–73. 10.1038/43441 10485706

[B26] Huang C. C. Hall D. H. Hedgecock E. M. Kao G. Karantza V. Vogel B. E.. (2003). Laminin alpha subunits and their role in *C. elegans* development. Development 130, 3343–3358. 10.1242/dev.00481 12783803

[B27] Kalil K. Dent E. W. (2005). Touch and go: guidance cues signal to the growth cone cytoskeleton. Curr. Opin. Neurobiol. 15, 521–526. 10.1016/j.conb.2005.08.005 16143510

[B28] Karlstrom R. O. Trowe T. Klostermann S. Baier H. Brand M. Crawford A. D.. (1996). Zebrafish mutations affecting retinotectal axon pathfinding. Development 123, 427–438. 9007260 10.1242/dev.123.1.427

[B29] Kizuka Y. Matsui T. Takematsu H. Kozutsumi Y. Kawasaki T. Oka S. (2006). Physical and functional association of glucuronyltransferases and sulfotransferase involved in HNK-1 biosynthesis. J. Biol. Chem. 281, 13644–13651. 10.1074/jbc.M601453200 16543228

[B30] Kuhn T. B. Schmidt M. F. Kater S. B. (1995). Laminin and fibronectin guideposts signal sustained but opposite effects to passing growth cones. Neuron 14, 275–285. 10.1016/0896-6273(95)90285-6 7531986

[B31] Kury P. Zickler P. Stoll G. Hartung H. P. Jander S. (2005). Osteopontin, a macrophage-derived matricellular glycoprotein, inhibits axon outgrowth. FASEB J. 19, 398–400. 10.1096/fj.04-1777fje 15625076

[B32] Lathia J. D. Patton B. Eckley D. M. Magnus T. Mughal M. R. Sasaki T.. (2007). Patterns of laminins and integrins in the embryonic ventricular zone of the CNS. J. Comp. Neurol. 505, 630–643. 10.1002/cne.21520 17948866

[B33] Liaw L. Skinner M. P. Raines E. W. Ross R. Cheresh D. A. Schwartz S. M.. (1995). The adhesive and migratory effects of osteopontin are mediated via distinct cell surface integrins. Role of alpha v beta 3 in smooth muscle cell migration to osteopontin *in vitro*. J. Clin. Invest. 95, 713–724. 10.1172/JCI117718 7532190 PMC295539

[B34] Liesi P. Narvanen A. Soos J. Sariola H. Snounou G. (1989). Identification of a neurite outgrowth-promoting domain of laminin using synthetic peptides. FEBS Lett. 244, 141–148. 10.1016/0014-5793(89)81180-9 2924902

[B35] Louvi A. Wassef M. (2000). Ectopic engrailed 1 expression in the dorsal midline causes cell death, abnormal differentiation of circumventricular organs and errors in axonal pathfinding. Development 127, 4061–4071. 10952903 10.1242/dev.127.18.4061

[B36] Mastick G. S. Easter S. S. Jr. (1996). Initial organization of neurons and tracts in the embryonic mouse fore- and midbrain. Dev. Biol. 173, 79–94. 10.1006/dbio.1996.0008 8575640

[B37] Merchan P. Bardet S. M. Puelles L. Ferran J. L. (2011). Comparison of pretectal genoarchitectonic pattern between quail and chicken embryos. Front. Neuroanat. 5:23. 10.3389/fnana.2011.00023 21503155 PMC3074437

[B38] Milner R. Campbell I. L. (2002). The integrin family of cell adhesion molecules has multiple functions within the CNS. J. Neurosci. Res. 69, 286–291. 10.1002/jnr.10321 12125070

[B39] Morita I. Kakuda S. Takeuchi Y. Kawasaki T. Oka S. (2009). HNK-1 (human natural killer-1) glyco-epitope is essential for normal spine morphogenesis in developing hippocampal neurons. Neuroscience 164, 1685–1694. 10.1016/j.neuroscience.2009.09.065 19796667

[B40] Myers J. P. Santiago-Medina M. Gomez T. M. (2011). Regulation of axonal outgrowth and pathfinding by integrin-ECM interactions. Dev. Neurobiol. 71, 901–923. 10.1002/dneu.20931 21714101 PMC3192254

[B41] Nakamoto T. Kain K. H. Ginsberg M. H. (2004). Neurobiology: new connections between integrins and axon guidance. Curr. Biol. 14, R121–R123. 10.1016/j.cub.2004.01.020 14986683

[B42] Nakamura R. Nakamura F. Fukunaga S. (2015). Diverse functions of perlecan in central nervous system cells *in vitro*. Anim. Sci. J. 86, 904–911. 10.1111/asj.12376 25781054

[B43] Nawabi H. Castellani V. (2011). Axonal commissures in the central nervous system: how to cross the midline? Cell. Mol. Life Sci. 68, 2539–2553. 10.1007/s00018-011-0691-9 21538161 PMC11114790

[B44] Ong E. Suzuki M. Belot F. Yeh J. C. Franceschini I. Angata K.. (2002). Biosynthesis of HNK-1 glycans on O-linked oligosaccharides attached to the neural cell adhesion molecule (NCAM): the requirement for core 2 beta 1,6-N-acetylglucosaminyltransferase and the muscle-specific domain in NCAM. J. Biol. Chem. 277, 18182–18190. 10.1074/jbc.M201312200 11891229

[B45] Paulus J. D. Halloran M. C. (2006). Zebrafish bashful/laminin-alpha 1 mutants exhibit multiple axon guidance defects. Dev. Dyn. 235, 213–224. 10.1002/dvdy.20604 16261616

[B46] Plantman S. (2012). Osteopontin is upregulated after mechanical brain injury and stimulates neurite growth from hippocampal neurons through beta1 integrin and CD44. Neuroreport 23, 647–652. 10.1097/WNR.0b013e328355380e 22692550

[B47] Puelles L. Rubenstein J. L. (2003). Forebrain gene expression domains and the evolving prosomeric model. Trends Neurosci. 26, 469–476. 10.1016/S0166-2236(03)00234-0 12948657

[B48] Ramos C. Fernandez-Llebrez P. Bach A. Robert B. Soriano E. (2004). Msx1 disruption leads to diencephalon defects and hydrocephalus. Dev. Dyn. 230, 446–460. 10.1002/dvdy.20070 15188430

[B49] Ren T. van der Merwe Y. Steketee M. B. (2015). Developing extracellular matrix technology to treat retinal or optic nerve injury. eNeuro 2, 1–14. 10.1523/ENEURO.0077-15.2015 26478910 PMC4603254

[B50] Ries A. Goldberg J. L. Grimpe B. (2007). A novel biological function for CD44 in axon growth of retinal ganglion cells identified by a bioinformatics approach. J. Neurochem. 103, 1491–1505. 10.1111/j.1471-4159.2007.04858.x 17760872 PMC2901540

[B51] Schoebitz K. Garrido O. Heinrichs M. Speer L. Rodriguez E. M. (1986). Ontogenetical development of the chick and duck subcommissural organ. An immunocytochemical study. Histochemistry 84, 31–40. 10.1007/BF00493417 2420757

[B52] Scholzen T. Solursh M. Suzuki S. Reiter R. Morgan J. L. Buchberg A. M.. (1994). The murine decorin. Complete cDNA cloning, genomic organization, chromosomal assignment, and expression during organogenesis and tissue differentiation. J. Biol. Chem. 269, 28270–28281. 7961765

[B53] Schwarz M. Alvarez-Bolado G. Dressler G. Urbanek P. Busslinger M. Gruss P. (1999). Pax2/5 and Pax6 subdivide the early neural tube into three domains. Mech. Dev. 82, 29–39. 10.1016/S0925-4773(99)00005-2 10354469

[B54] Senn C. Kutsche M. Saghatelyan A. Bosl M. R. Lohler J. Bartsch U.. (2002). Mice deficient for the HNK-1 sulfotransferase show alterations in synaptic efficacy and spatial learning and memory. Mol. Cell. Neurosci. 20, 712–729. 10.1006/mcne.2002.1142 12213450

[B55] Shay E. L. Greer C. A. Treloar H. B. (2008). Dynamic expression patterns of ECM molecules in the developing mouse olfactory pathway. Dev. Dyn. 237, 1837–1850. 10.1002/dvdy.21595 18570250 PMC2787191

[B56] Smith L. L. Cheung H. K. Ling L. E. Chen J. Sheppard D. Pytela R.. (1996). Osteopontin N-terminal domain contains a cryptic adhesive sequence recognized by alpha9beta1 integrin. J. Biol. Chem. 271, 28485–28491. 10.1074/jbc.271.45.28485 8910476

[B57] Soulintzi N. Zagris N. (2007). Spatial and temporal expression of perlecan in the early chick embryo. Cells Tissues Organs 186, 243–256. 10.1159/000107948 17785960

[B58] Stanic K. Montecinos H. Caprile T. (2010). Subdivisions of chick diencephalic roof plate: implication in the formation of the posterior commissure. Dev. Dyn. 239, 2584–2593. 10.1002/dvdy.22387 20730872

[B59] Stanic K. Vera A. Gonzalez M. Recabal A. Astuya A. Torrejon M.. (2014). Complementary expression of EphA7 and SCO-spondin during posterior commissure development. Front. Neuroanat. 8:49. 10.3389/fnana.2014.00049 25009468 PMC4068196

[B60] Suh L. H. Oster S. F. Soehrman S. S. Grenningloh G. Sretavan D. W. (2004). L1/Laminin modulation of growth cone response to EphB triggers growth pauses and regulates the microtubule destabilizing protein SCG10. J. Neurosci. 24, 1976–1986. 10.1523/JNEUROSCI.1670-03.2004 14985440 PMC6730397

[B61] Tosa Y. Tsukano K. Itoyama T. Fukagawa M. Nii Y. Ishikawa R.. (2015). Involvement of Slit-Robo signaling in the development of the posterior commissure and concomitant swimming behavior in *Xenopus laevis*. Zoological Lett. 1, 28. 10.1186/s40851-015-0029-9 26605073 PMC4657333

[B62] Turney S. G. Bridgman P. C. (2005). Laminin stimulates and guides axonal outgrowth via growth cone myosin II activity. Nat. Neurosci. 8, 717–719. 10.1038/nn1466 15880105

[B63] Ware M. Dupe V. Schubert F. R. (2015). Evolutionary conservation of the early axon scaffold in the vertebrate brain. Dev. Dyn. 244, 1202–1214. 10.1002/dvdy.24312 26228689

[B64] Ware M. Schubert F. R. (2011). Development of the early axon scaffold in the rostral brain of the chick embryo. J. Anat. 219, 203–216. 10.1111/j.1469-7580.2011.01389.x 21599661 PMC3162240

[B65] Weinl C. Drescher U. Lang S. Bonhoeffer F. Loschinger J. (2003). On the turning of Xenopus retinal axons induced by ephrin-A5. Development 130, 1635–1643. 10.1242/dev.00386 12620987

[B66] Westerfield M. (1987). Substrate interactions affecting motor growth cone guidance during development and regeneration. J. Exp. Biol. 132, 161–175. 3323398 10.1242/jeb.132.1.161

[B67] Wright M. C. Mi R. Connor E. Reed N. Vyas A. Alspalter M.. (2014). Novel roles for osteopontin and clusterin in peripheral motor and sensory axon regeneration. J. Neurosci. 34, 1689–1700. 10.1523/JNEUROSCI.3822-13.2014 24478351 PMC3905142

[B68] Yagi H. Yanagisawa M. Suzuki Y. Nakatani Y. Ariga T. Kato K.. (2010). HNK-1 epitope-carrying tenascin-C spliced variant regulates the proliferation of mouse embryonic neural stem cells. J. Biol. Chem. 285, 37293–37301. 10.1074/jbc.M110.157081 20855890 PMC2988335

[B69] Yamamoto S. Oka S. Inoue M. Shimuta M. Manabe T. Takahashi H.. (2002). Mice deficient in nervous system-specific carbohydrate epitope HNK-1 exhibit impaired synaptic plasticity and spatial learning. J. Biol. Chem. 277, 27227–27231. 10.1074/jbc.C200296200 12032138

[B70] Zimmermann D. R. Dours-Zimmermann M. T. (2008). Extracellular matrix of the central nervous system: from neglect to challenge. Histochem. Cell Biol. 130, 635–653. 10.1007/s00418-008-0485-9 18696101

