# Supplementary figures and images for: Expression Patterns of Extracellular Matrix Proteins during Posterior Commissure Development
Source: Front Neuroanat. 2016 Sep 28;10:89. doi: 10.3389/fnana.2016.00089 (PMC5039192; doi:10.3389/fnana.2016.00089)

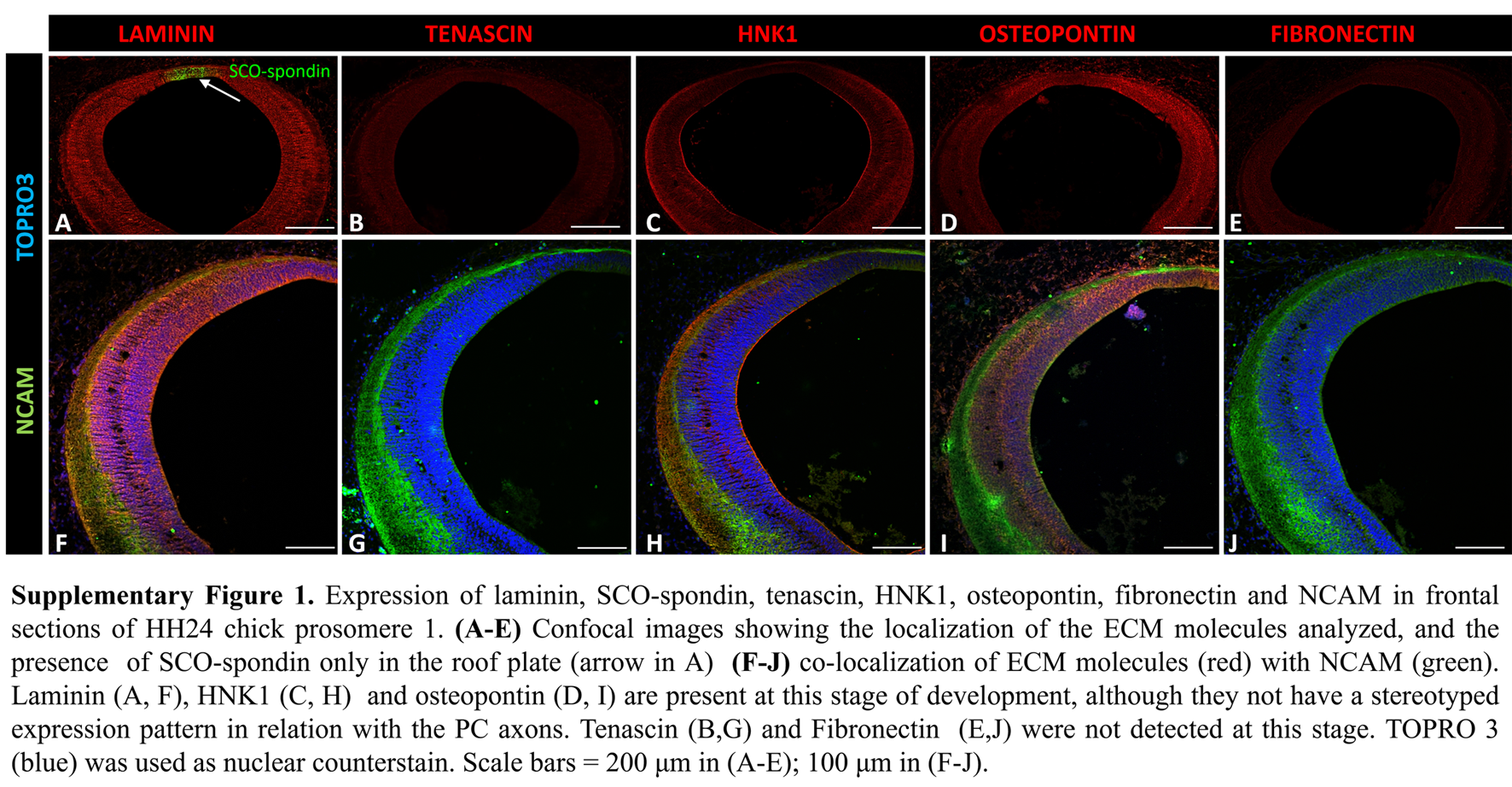

Supplement: Supplementary file 1 [file Image1.TIF]
